# ATF5 Attenuates the Secretion of Pro-Inflammatory Cytokines in Activated Microglia

**DOI:** 10.3390/ijms24043322

**Published:** 2023-02-07

**Authors:** Jiebo Zhu, Min Joung Lee, Jong Hun An, Eungseok Oh, Woosuk Chung, Jun Young Heo

**Affiliations:** 1 Department of Medical Science, Chungnam National University School of Medicine, Daejeon 35015, Republic of Korea; 2 Department of Biochemistry, Chungnam National University School of Medicine, Daejeon 35015, Republic of Korea; 3 Infection Control Convergence Research Center, Chungnam National University School of Medicine, Daejeon 35015, Republic of Korea; 4 Department of Neurology, Chungnam National University Hospital, Daejeon 35015, Republic of Korea; 5 Department of Anesthesiology and Pain Medicine, Chungnam National University School of Medicine, Daejeon 35015, Republic of Korea; 6 Department of Anesthesiology and Pain Medicine, Chungnam National University Hospital, Daejeon 35015, Republic of Korea

**Keywords:** ATF5, microglia, pro-inflammatory cytokines, MMP

## Abstract

The highly dynamic changes in microglia necessary to achieve a rapid neuroinflammatory response require a supply of energy from mitochondrial respiration, which leads to the accumulation of unfolded mitochondrial proteins. We previously reported that microglial activation is correlated with the mitochondrial unfolded protein response (UPRmt) in a kaolin-induced hydrocephalus model, but we still do not know the extent to which these changes in microglia are involved in cytokine release. Here, we investigated the activation of BV-2 cells and found that treatment with lipopolysaccharide (LPS) for 48 h increased the secretion of pro-inflammatory cytokines. This increase was accompanied by a concurrent decrease in oxygen consumption rate (OCR) and mitochondrial membrane potential (MMP), in association with the up-regulation of the UPRmt. Inhibition of the UPRmt by knockdown of ATF5, a key upstream regulator of the UPRmt, using small-interfering RNA against ATF5 (siATF5) not only increased production of the pro-inflammatory cytokines, interleukin-6 (IL-6), IL-1β and tumor necrosis factor-α (TNF-α), but also decreased MMP. Our results suggest that ATF5-dependent induction of the UPRmt in microglia acts as a protective mechanism during neuroinflammation and may be a potential therapeutic target for reducing neuroinflammation.

## 1. Introduction

Microglia, regarded as neuroinflammatory sentinel cells, are widely distributed in brain tissue, where they act as the first line of defense against inflammation and pathological injury in the central nervous system (CNS) [1]. Activated microglia and the associated induction of proinflammatory cytokines are common features in acute brain injury, including traumatic brain injury (TBI), intracerebral hemorrhage (ICH), and subarachnoid hemorrhage (SAH) [2,3,4]. According to recent reports, mitochondria act as important regulators of microglia-induced inflammatory responses [5]. Mitochondria-derived damage-associated molecular patterns (DAMPs) from neurons, such as transcription factor A mitochondria (TFAM), adenosine triphosphate (ATP) and cytochrome *c*, act on immune receptors of glia, accelerating the production of the pro-inflammatory cytokines IL-6, IL-1β, and TNF-α in a non-cell-autonomous manner [6,7]. Microglia are also activated via a cell-autonomous mechanism that is associated with the reprogramming of mitochondrial metabolism, specifically the conversion from oxidative phosphorylation (OXPHOS) to glycolysis, which serves to meet the need for a high rate of ATP production [8]. After recovering from inflammatory stimulation, mitochondrial metabolism reverts to OXPHOS and activated microglia return to a resting state. However, lipopolysaccharide (LPS) toxicity, which is well known to cause microglial activation, is accompanied by mitochondrial dysfunction and rapidly (~2.5 h) upregulates microglial glycolysis; notably, the glycolysis inhibitors 2-deoxy-D-glucose (2-DG) and 3-bromopyruvate (3-BPA) reduce LPS-induced secretion of the pro-inflammatory cytokines IL-6 and TNF-*α* [9]. Although non-cell- and cell-autonomous regulation of microglia through changes in mitochondrial function has been demonstrated, how mitochondrial dysfunction is linked to cytokine production in microglia remains unclear.

Dysfunctional mitochondria generate reactive oxygen species (ROS) and misfolded and unfolded proteins, which trigger mitochondrial stress responses [10,11]. The mitochondrial unfolded protein response (UPRmt) is a cellular adaptation process initiated by the accumulation of damaged proteins that are not removed as a result of mitochondrial dysfunction [12]. Mitochondrial stress triggers the translocation of activating transcription factor 5 (ATF5) from mitochondria into the nucleus [13]. There, ATF5 induces the expression of UPRmt-related molecular chaperones and proteases such as heat shock protein 60 (HSP60), mitochondrial protease Lon protease (LONP1) and caseinolytic peptidase P (CLPP), which interact with unfolded and misfolded proteins to improve their folding ability or degrade them [14]. During the response to an inflammatory microenvironment, ATF5 stimulates UPRmt activity, thereby promoting the recovery of damaged mitochondria and leading to enhanced innate immunity [6]. LPS also activates the UPRmt in cardiomyocytes, which act to reduce inflammation-mediated myocardial injury [15]. We previously reported that microglia are activated and the UPRmt is upregulated in a mouse model of kaolin-induced hydrocephalus [16]. However, the potential link between microglia-induced neuroinflammation and the UPRmt has not been well studied. In the current study, we hypothesized that ATF5 regulates microglial activation and is involved in releasing proinflammatory cytokines. Starting from the observation that LPS treatment induces ATF5 expression and upregulates the UPRmt, we assessed changes in inflammatory cytokines and mitochondrial function induced by the knockdown of ATF5 with small interfering RNA (siATF5). This study provides novel insights into the potential mechanism underlying microglial activation and cytokine induction after mitochondrial stress, highlighting a previously unappreciated role of ATF5.

## 2. Results

### 2.1. LPS Treatment Upregulates Pro-Inflammatory Cytokines and the UPRmt in BV-2 Cells

**Figure 1:**
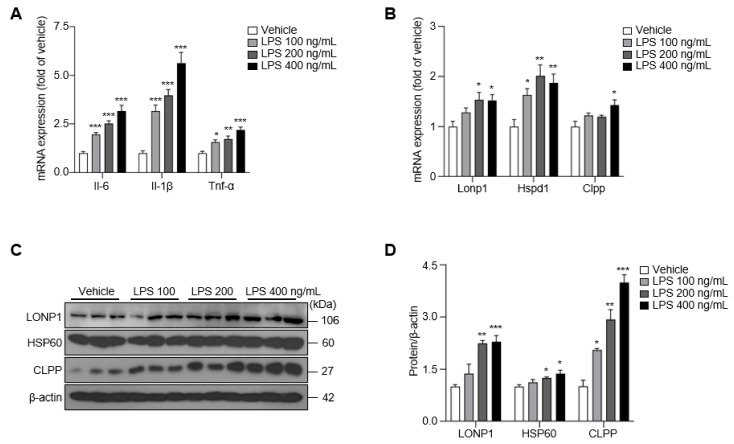
The expression of pro-inflammatory cytokines and UPRmt was upregulated in LPS-treated BV-2 cells for 48 h. (**A**) The expression of *Il-6*, *Il-1β*, and *Tnf-α* in LPS-treated BV-2 cells. (**B**) The expression of *Lonp1*, *Hspd1*, and *Clpp* in LPS-treated BV-2 cells. (**C**) The expression of LONP1, HSP60, and CLPP in LPS-treated BV-2 cells. (**D**) The intensity value of LONP1, HSP60, and CLPP was shown. Data are presented as mean ± SEM of three independent experiments (* *p* < 0.05, ** *p* < 0.01, *** *p* < 0.001 compared to vehicle).

The increase in misfolded and unfolded proteins induced by LPS treatment activates the UPRmt, which promotes the recovery of mitochondrial function [17]. To assess the involvement of the UPRmt in LPS-induced microglial inflammation, we first examined the mRNA levels of several pro-inflammatory cytokines, mitochondrial proteases and chaperones in BV-2 cells after LPS treatment. Both mRNAs levels of pro-inflammatory cytokines and UPRmt activity were unchanged at 6 and 12 h compared with the vehicle control group (Figure S1A,B). In contrast, mRNA levels of the pro-inflammatory cytokines (*Il-6*, *Il-1β* and *Tnf-α*), *Atf5, Lonp1*, *Hspd1* and *Clpp*, showed significant upregulation at 24 h (Figure S1C), although this change did not translate to an increase in expression of the corresponding proteins at this timepoint (Figure S1D,E). After treatment of BV-2 cells with LPS for 48 h, *Il-6* and *Tnf-α* mRNA expression was increased by ~2.5-fold and *Il-1β* mRNA expression was increased by more than 3-fold compared with that in the vehicle group (Figure 1A). *Lonp1*, *Hspd1* and *Clpp* mRNA expression was also increased (by ~1.5-fold) at this time point compared with the vehicle group (Figure 1B), increases that were associated with the prominent upregulation of the protein levels of LONP1, HSP60, and CLPP (Figure 1C,D). These results support the conclusion that LPS treatment induces mRNA expression of pro-inflammatory cytokines and upregulates the UPRmt at 24 h in BV-2 cells, effects that were increased at the translational level at 48 h. On the basis of these results, we used a 48 h LPS treatment paradigm for subsequent experiments.

### 2.2. LPS Slightly Deteriorates Mitochondrial Function in BV-2 Cells

**Figure 2:**
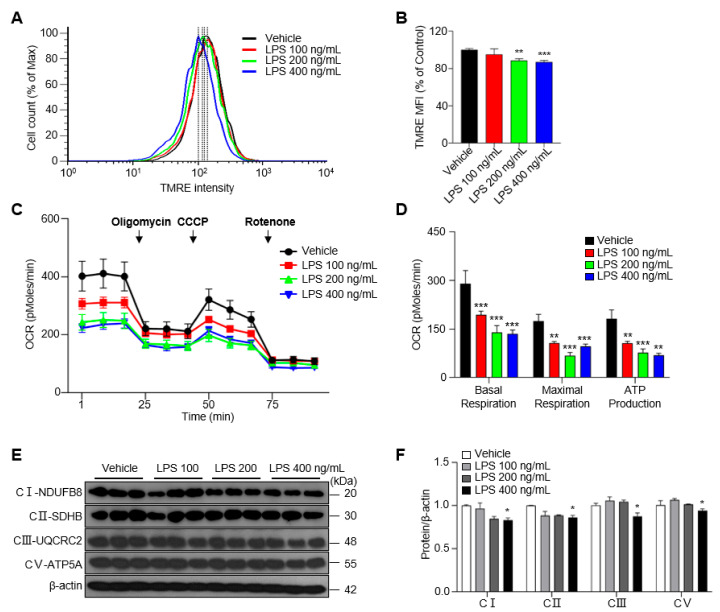
Treatment of LPS decreases the mitochondrial membrane potential (MMP) and mitochondrial respiration in BV-2 cells for 48 h. (**A**) The MMP determined by TMRE-stained BV-2 cells. (**B**) Median fluorescence intensity (MFI) values as analyzed by the FlowJo program. (**C**) Oxygen consumption rate (OCR) measured in BV-2 cells treated with vehicle or LPS at 48 h. (**D**) The basal respiration, maximal respiration, and ATP production calculated by means of XF24 analyzer software. (**E**) The expression of mitochondrial complexes Ⅰ, Ⅱ, Ⅲ and Ⅴ in LPS-treated BV-2 cells. (**F**) The intensity values of mitochondrial complexes are shown. Data are presented as mean ± SEM of three independent experiments (* *p* < 0.05, ** *p* < 0.01, *** *p* < 0.001 compared to vehicle).

Microglial activation is associated with alterations in mitochondrial metabolism in neurodegeneration [18]. Mitochondrial membrane potential (MMP)—the electron transfer potential across the mitochondrial inner membrane—is key to the operation of the mitochondrial OXPHOS system, which is needed for ATP production [19]. To investigate whether MMP is influenced by LPS treatment, we measured the median fluorescence intensity (MFI) of the membrane potential-sensitive dye TMRE (tetramethylrhodamine ethyl ester) by flow cytometry. We found that the treatment of BV-2 cells with LPS at concentrations of 200 or 400 ng/mL for 48 h reduced the MFI of TMRE by ~10% compared with the vehicle control group (Figure 2A,B). To further identify functional changes in OXPHOS in activated microglia, we measured the oxygen consumption rate (OCR) in LPS-treated BV-2 cells. Basal respiration, maximal respiration, and ATP production were decreased by LPS treatment compared with those in the vehicle control group (Figure 2C,D). Consistent with this, the expression of proteins composing mitochondrial complexes Ⅰ, Ⅱ, Ⅲ and Ⅴ responsible for mitochondrial OXPHOS decreased after treatment with 400 ng/mL LPS (Figure 2E,F). Taken together, these results suggest that LPS treatment modestly reduces mitochondrial OXPHOS function in BV-2 cells, even with a long exposure time.

### 2.3. siRNA-Mediated Knockdown of ATF5 Reduces the UPRmt in Microglia

**Figure 3:**
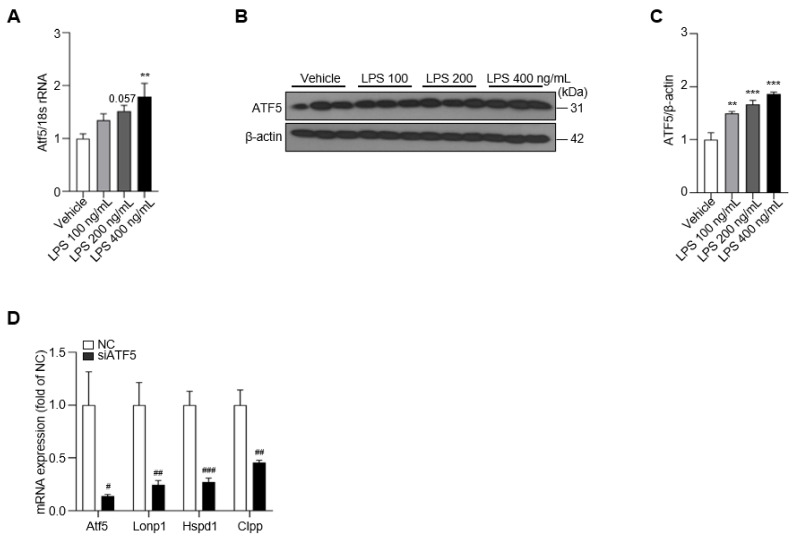
Treatment of LPS increases the level of ATF5, which can be silenced by siATF5. (**A**) The expression of *Atf5* is upregulated in LPS-treated BV-2 cells at 48 h. (**B**) The protein level of ATF5 determined in LPS-treated BV-2 cells for 48 h. (**C**) The intensity value of ATF5 is shown. (**D**) The expression of *Atf5*, *Lonp1*, *Hspd1*, and *Clpp* in NC- or siATF5-treated BV-2 cells for 24 h. Data are presented as mean ± SEM of three independent experiments (** *p* < 0.01, *** *p* < 0.001 compared to vehicle, # *p* < 0.05, ## *p* < 0.01, ### *p* < 0.001 compared to NC).

UPRmt activation is a consequence of intracellular mitochondrial stimulation in the context of neuroinflammatory stress [20]. However, how the UPRmt is regulated in LPS-treated BV-2 cells and what role this activation plays remain unknown. To address this, we first assessed the expression of ATF5, which is involved in the activation of the UPRmt, following LPS treatment of BV-2 cells [13]. We found that ATF5 mRNA and protein expression were increased by ~2-fold in the LPS-treated group compared with the vehicle control group (Figure 3A–C). To ascertain the role of ATF5 in the UPRmt in BV-2 cells, we knocked down the expression of ATF5 using small-interfering RNA targeting ATF5 (siATF5). As expected, siATF5 significantly decreased the expression of *Atf5*, *Lonp1*, *Hspd1* and *Clpp* compared with scrambled, negative control (NC) siRNA (Figure 3D). Moreover, siATF5 reduced the levels of the *Atf5* transcript, even after LPS treatment (Figure S2). These results suggest that the intracellular ATF5–UPRmt axis is activated by LPS treatment in BV-2 cells, an effect that is inhibited by knocking down ATF5.

### 2.4. ATF5 Attenuates the Release of Proinflammatory Cytokines

**Figure 4:**
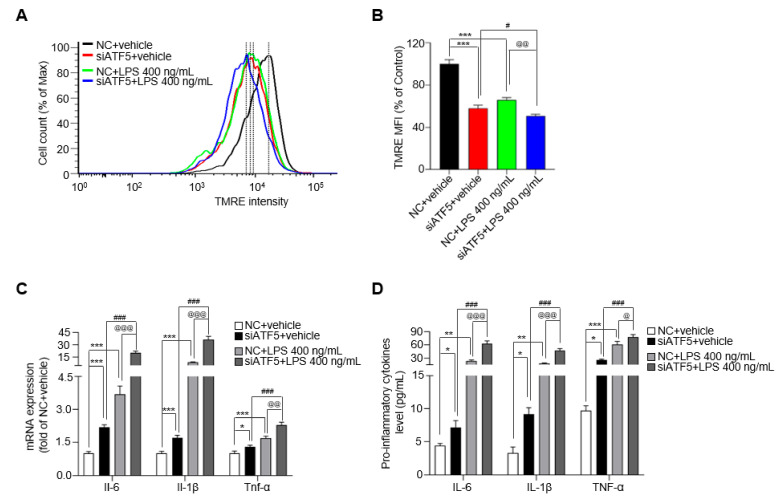
siATF5 upregulates the secretion of pro-inflammatory cytokines with downregulates mitochondrial membrane potential (MMP). (**A**) The MMP determined by TMRE-stained BV-2 cells. (**B**) Median fluorescence intensity (MFI) values analyzed by the FlowJo 7.6 program. (**C**) The expression of *Il-6*, *Il-1β*, and *Tnf-α* in NC- or siATF5-treated for 24 h and vehicle- or LPS-treated BV-2 cells for another 48 h. (**D**) The protein level of IL-6, IL-1β, and TNF-α as determined by ELISA. Data are presented as mean ± SEM of three independent experiments (* *p* < 0.05, ** *p* < 0.01, *** *p* < 0.001 compared to NC+vehicle, # *p* < 0.05, ### *p* < 0.001 compared to siATF5+vehicle, @ *p* < 0.05, @@ *p* < 0.01, @@@ *p* < 0.001 compared to NC+LPS 400 ng/mL).

ATF5 is a transcription factor that is induced by mitochondrial stress in mammalian cells and is essential for cellular responses to mitochondrial dysfunction [20]. To establish the relationship between ATF5 and mitochondrial function in microglia, we measured OCR and MMP after ATF5 knockdown in BV-2 cells. The basal respiration and maximal respiration were decreased by over 1.5-fold after treatment with siATF5 in BV-2 cells compared with the NC group, and ATP production was reduced by 1.1-fold after treatment with siATF5 in BV-2 cells compared with the NC group for 24 h (Figure S3A,B). In addition, the expression of mitochondrial complexes Ⅱ, Ⅲ and Ⅴ was down-regulated by ~20% after treatment with siATF5 compared with the NC group (Figure S3C,D). As expected, LPS reduced MMP in NC cells, reducing it to 65.8% of that in NC+vehicle-treated cells (Figure 4A,B). Interestingly, siATF5+vehicle-treated cells reduced MMP to 57.9% of that in NC+vehicle-treated cells, and the MMP showed more reduction (by 15.1%) in siATF5+LPS-treated cells than in NC+LPS-treated cells (Figure 4A,B). Given that ATF5 mediates inflammatory responses induced by mitochondrial stress, it could be involved in the regulation of cytokine release in BV-2 cells. To identify changes in cytokine release induced by ATF5, we assessed cytokine expression levels following LPS treatment in ATF5-knockdown BV-2 cells. *Il-6* mRNA levels were increased by 3.7-fold and *Il-1β* and *Tnf-α* levels were increased by ~1.5-fold in the NC+LPS group compared with the NC+vehicle group (Figure 4C). In addition, *Il-6* and *Il-1β* mRNA levels were more than 9-fold higher in siATF5+LPS-treated cells than in NC+LPS-treated cells, and *Tnf-α* levels were 1.8-fold higher (Figure 4C), representing significant differences. Consistent with changes in mRNA levels, the release of IL-6 and IL-1β proteins increased by ~2.5 fold in the siATF5+LPS group compared with the NC+LPS group, whereas the release of TNF-α increased by ~1.3 fold, as determined by enzyme-linked immunosorbent assay (ELISA) analysis of cell culture media (Figure 4D). These results show that the upregulation of ATF5 by mitochondrial dysfunction mitigates LPS-induced secretion of pro-inflammatory cytokines in BV-2 cells.

## 3. Discussion

Microglia are responsible for scavenging damaged cells and mediating neuroinflammation in the CNS, playing an essential role in CNS homeostasis [21]. To accomplish their functions, microglia consume substantial amounts of energy, a heightened demand that is met by an increase in the bioenergetic metabolism of mitochondria [22]. We previously reported that activated microglia and the UPRmt (reflecting mitochondrial stress) are involved in the pathogenic mechanism underlying hydrocephalus in a kaolin-induced hydrocephalus mouse model [16]. To further establish the relationship between activated microglia and the UPRmt, we analyzed the microglia-mediated inflammatory response, UPRmt and mitochondrial OXPHOS function in LPS-stimulated BV-2 cells. We found that LPS treatment of BV-2 cells for 48 h increased the production of the pro-inflammatory cytokines, *Il-6*, *Il-1β* and *Tnf-α*, and increased expression of the UPRmt molecules, LONP1, HSP60 and CLPP, in association with mitochondrial dysfunction. Interestingly, siRNA-mediated knockdown of the UPRmt upstream regulator, ATF5, further increased the expression and release of pro-inflammatory cytokines in microglia.

The role of mitochondrial homeostasis in neuroinflammatory responses has gradually come into focus [23]. The UPRmt-related molecules HSP60, LONP1 and CLPP respond to neuroinflammatory stimuli and have been shown to exert protective effects against brain injury. Honokiol, extracted from the bark of *Magnolia officinalis*, inhibits the secretion of pro-inflammatory cytokines in a rat TBI model by upregulating the expression of HSP60 and CLPP [24]. In the N9 microglial cells, the pro-inflammatory cytokine IL-1β induces the production of HSP60, which binds to the Toll-like receptor 4 (TLR4) of N9 microglial cells, further inhibiting the secretion of IL-1β to suppress the inflammatory response [25]. Propionic acid (PPA) exerts a neuroprotective effect against ischemic brain injury that is associated with the upregulation of LONP1 [26]. In the current study, we found that LPS significantly increased expression of the pro-inflammatory cytokines *Il-6*, *Il-1β* and *Tnf-α*, in association with the up-regulation of LONP1, HSP60 and CLPP. Notably, the expression of CLPP protein was increased by more than 4-fold (to 400 ng/mL) in the LPS-treated group compared with the vehicle control group. It has been reported that CLPP functions in the stabilization of the mitochondrial OXPHOS complex in the forebrain [27]. Moreover, CLPP is helpful in reducing αSyn-induced mitochondrial oxidative stress in Parkinson’s patients by increasing the expression of superoxide dismutase-2 (SOD2) [28]. There is limited evidence for a role for CLPP in neuroinflammation in glial cells; however, based on our results showing LPS stimulation-induced changes in CLPP, which showed the highest expression among UPRmt-related proteins, we could expect CLPP to have an important role in neuroinflammation, a question that warrants further investigation in vivo using glial cell type-specific CLPP-modulated transgenic mice.

Mitochondrial quality control (QC) processes, which include mitophagy, the UPRmt and the ubiquitination of damaged mitochondrial proteins [17], enhance intracellular resilience capacity against continuous, low-grade, chronic inflammatory stress [29]. The metabolic state of LPS-activated microglia shifts from OXPHOS to glycolysis, reflecting the beneficial effect of more rapid energy production from glycolysis on the production of proinflammatory cytokines [9,18,30,31]. In keeping with this mitochondrial metabolic reprogramming, previous reports found slight mitochondrial OXPHOS defects together with decreased MMP in LPS-activated microglia [32,33]. In the current study, we did not further verify the role of other mitochondrial QC systems, such as mitophagy, mitochondrial dynamics and ubiquitination. However, there are several reports indicating that these mitochondrial QC systems contribute to neuroinflammation responses. The mitochondria-regulating proteins, parkin and DRP1, which are associated with mitophagy and mitochondrial fission, respectively, are involved in the suppression of LPS-induced microglial neuroinflammation [34,35,36]. Ubiquitin-specific protease 7 (USP7), which is essential for mediating protein homeostasis, significantly attenuates microglial neuroinflammation [37]. In addition, deletion of the ubiquitin-editing enzyme, A20/TNF-α induced protein 3 (TNFAIP3), in the microglia enhances the response to LPS-induced neuroinflammation [38]. Resilience against mitochondrial dysfunction in the context of LPS toxicity might be associated with mitochondrial molecular-level QC processes, such as the UPRmt, but additional investigation is still required to determine how these processes affect cellular metabolic reprogramming and homeostasis during neuroinflammation and identify the underlying molecular mechanism.

ATF5 acts as an upstream regulator to mediate the mitochondrial integrated stress response (ISR), which is involved in the cellular stress response pathway [39]. The ATF5–UPRmt axis contributes to the repair of damaged mitochondria and maintains protein homeostasis [40]. Consistent with these reports, we found that the expression of ATF5 and UPRmt-related molecules LONP1, HSP60 and CLPP increased after LPS treatment. Mitochondria sense pro-inflammatory responses and play a crucial role in resistance to pathogenic stimuli [41]. Invading pathogens not only increase the expression of cytokines but also affect the correct folding of proteins, which suggests that targeting UPRmt–cytokine cross-talk is a potential direction for the treatment of inflammatory diseases [42]. In the current study, LPS-mediated inflammatory stimuli increased the expression of pro-inflammatory cytokine *Il-1β* in BV-2 cells from 12 h (Figure S1B), the mitochondrial stress response axis ATF5-UPRmt was activated in response to increased pro-inflammatory cytokines *Il-6*, *Il-1β* and *Tnf-α* at 24 h (Figure S1C). Consistent with our results, IL-1β increased ATF5 expression in HepG2 cells (human liver cancer cell line) in a dose-dependent manner; furthermore, knockdown of ATF5 in HepG2 cells resulted in increased IL-1β-induced expression of serum amyloid A protein 1 (SAA1) and SAA2, which are acute-phase isotypes produced in response to inflammatory stress [43]. Taken together, these observations suggest that ATF5 is induced by inflammatory response via UPRmt activation, further evaluation with the overexpression of ATF5 in BV-2 cells is required to identify the signaling pathway which affects the production of pro-inflammatory cytokines.

In addition, an ATF5 deficiency increased the production of pro-inflammatory cytokines in association with enhanced MMP depolarization compared with NC+vehicle treatment. This phenomenon was previously reported in ischemia reperfusion injury in the kidney, where the activated ATF5-mediated stress response pathway reduces the inflammatory response and improves tissue repair [44]. On the basis of these observations, we conclude that activation of ATF5 mitigates the progression of LPS-induced neuroinflammation in BV-2 cells. Notably, metformin and an extract of *Hibiscus syriacus* L. (HCE) suppress the production of pro-inflammatory cytokines in sepsis patients by maintaining MMP [45,46]. Maintenance of MMP by the antioxidant edaravone (EDA) is also involved in mitigating particulate matter (PM)-induced lung inflammation [47]. Moreover, by down-regulating secretion of the pro-inflammatory cytokines IL-6, IL-1β and TNF-α, and reversing the MMP, the peptide components of lymphocyte proliferation factor (LPF), GVYY and APTLW, purified from the low-molecular-weight fraction of walnut protein hydrolysate (WPHL), inhibit LPS-induced neuroinflammation in BV-2 cells [23]. Although these conclusions need to be verified in vivo, we would suggest that the regulation of ATF5 and the UPRmt could be candidates for attenuating the microglial activation and proinflammatory cytokine production that accompany mitochondrial dysfunction.

## 4. Materials and Methods

### 4.1. Cell Culture, Treatment, and Transfection

BV-2 murine microglial cells were maintained in a high-glucose medium (DMEM, Welgene, Gyeongsan-si, Korea) containing 10% FBS (Hyclone, Marlborough, MA, USA) and 1% penicillin/streptomycin (Hyclone, Marlborough, MA, USA) at 37 °C and 5% CO_2_. The BV-2 cells were activated with lipopolysaccharide (LPS) (Sigma-Aldrich, Louis, MO, USA) as reported previously, and phosphate-buffered saline (PBS) was used as the vehicle [48,49]. Additionally, the BV-2 cells were transfected with siATF5 (Thermo Scientific, #s200895, Carlsbad, CA, USA) and negative control (NC) siRNA (Bioneer, #SN-1002, Daejeon, Korea) using RNAimax (Thermo Scientific, #13778100, Carlsbad, CA, USA) for 24 h. After transfection, LPS (400 ng/mL) was treated to induce neuroinflammation for 48 h.

### 4.2. Quantitative Real-time PCR (qPCR)

Total RNAs were isolated using Trizol reagent (Thermo Fisher, Waltham, MA, USA) and cDNA was prepared from the total RNA with Reverse Transcription Master Premix (5×Rnase H+). The qPCR reaction was performed with cDNA, SYBR Green PCR Master Mix (PhileKorea, Seoul, Korea) and primers. Results were analyzed with the Rotor-Gene 6000 real-time rotary analyzer system (Corbett Life Science). For the primers used for qPCR, see Table S1.

### 4.3. Western Blotting

BV-2 cells were lysed using radioimmunoprecipitation assay (RIPA) buffer with phosphatase inhibitor and protease inhibitor cocktail (Roche) to extract the protein. The extracted protein underwent sodium dodecyl sulfate-polyacrylamide gel electrophoresis (SDS-PAGE) and then was transferred to a polyvinylidene fluoride (PVDF) membrane (Millipore). The PVDF membranes were blocked with 5% skim milk for 1 h, then incubated at 4 °C overnight. For the antibodies used for Western blotting, see Table S2.

### 4.4. Oxygen Consumption Rate (OCR)

BV-2 cells (3 × 10^3^/well) were incubated in media containing the vehicle, LPS NC or siATF5. Firstly, basal OCR was measured. Subsequently, ATPase inhibitor oligomycin A (20 µg/mL, Sigma-Aldrich, Louis, MO, USA), uncoupler carbonyl cyanide 3-chlorophenylhydrazone (CCCP, 50 µM, Sigma-Aldrich, Louis, MO, USA), and mitochondrial complex I inhibitor rotenone (20 µM, Sigma-Aldrich, Louis, MO, USA) were added and measured at 37 °C using an XF24 analyzer (Seahorse, North Billerica, MA, USA).

### 4.5. Mitochondrial Membrane Potential (MMP)

BV-2 cells (4 × 10^4^/well) were plated in media containing the vehicle or LPS. Mitochondrial membrane potential (MMP) was labeled using the tetramethylrhodamine ethyl ester (TMRE, 100 nM) at 37 °C for 20 min, washed with PBS, and centrifuged at 1000 rpm for 5 min at room temperature (RT). TMRE emits a red fluorescence that can be measured by flow cytometry on FACScan (BD Biosciences, Franklin Lakes, NJ, USA).

### 4.6. ELISA

The supernatants of LPS-treated BV-2 cells were centrifuged at 1500 rpm for 10 min at 4 °C. The supernatants were stored at −70 °C until the mouse IL-6, IL-1β, and TNF-α ELISA assays were performed (R&D system, #M6000B, #MLB00C, #MTA00B, Minneapolis, MN, USA) according to the manufacturer’s instructions.

### 4.7. Statistical Analysis

Statistical analysis of data was performed by means of GraphPad Prism 8 software (Graphpad, La Jolla, CA, USA) and data are presented as mean ± SEM. A two-tailed unpaired Student’s *t*-test, one-way ANOVA followed by Dunnett’s multiple comparisons test, and two-way ANOVA followed by Tukey’s multiple comparisons test were used for analyzing significance, and *p* < 0.05 was considered statistically significant.

## 5. Conclusions

LPS upregulates the expression of pro-inflammatory cytokines and the UPRmt but decreases mitochondrial OXPHOS and MMP in BV-2 cells. Knocking down ATF5, which acts upstream of the UPRmt, increases the expression of pro-inflammatory cytokines and causes the reduction in MMP expression. Taken together, our results suggest that, by virtue of its ability to maintain mitochondrial function, ATF5 is an important target for the treatment of microglial neuroinflammation.

## Data Availability

Data are included in the article.

## Supplementary Materials

The following supporting information can be downloaded at: https://www.mdpi.com/article/10.3390/ijms24043322/s1.
