# Supplementary material for: ATF5 Attenuates the Secretion of Pro-Inflammatory Cytokines in Activated Microglia
Source: Int J Mol Sci. 2023 Feb 7;24(4):3322. doi: 10.3390/ijms24043322 (PMC9961550; doi:10.3390/ijms24043322)
Supplement: Supplementary file 1 [file ijms-24-03322-s001.zip › ijms-2188685-supplementary.pdf]

Supplementary Figure S1

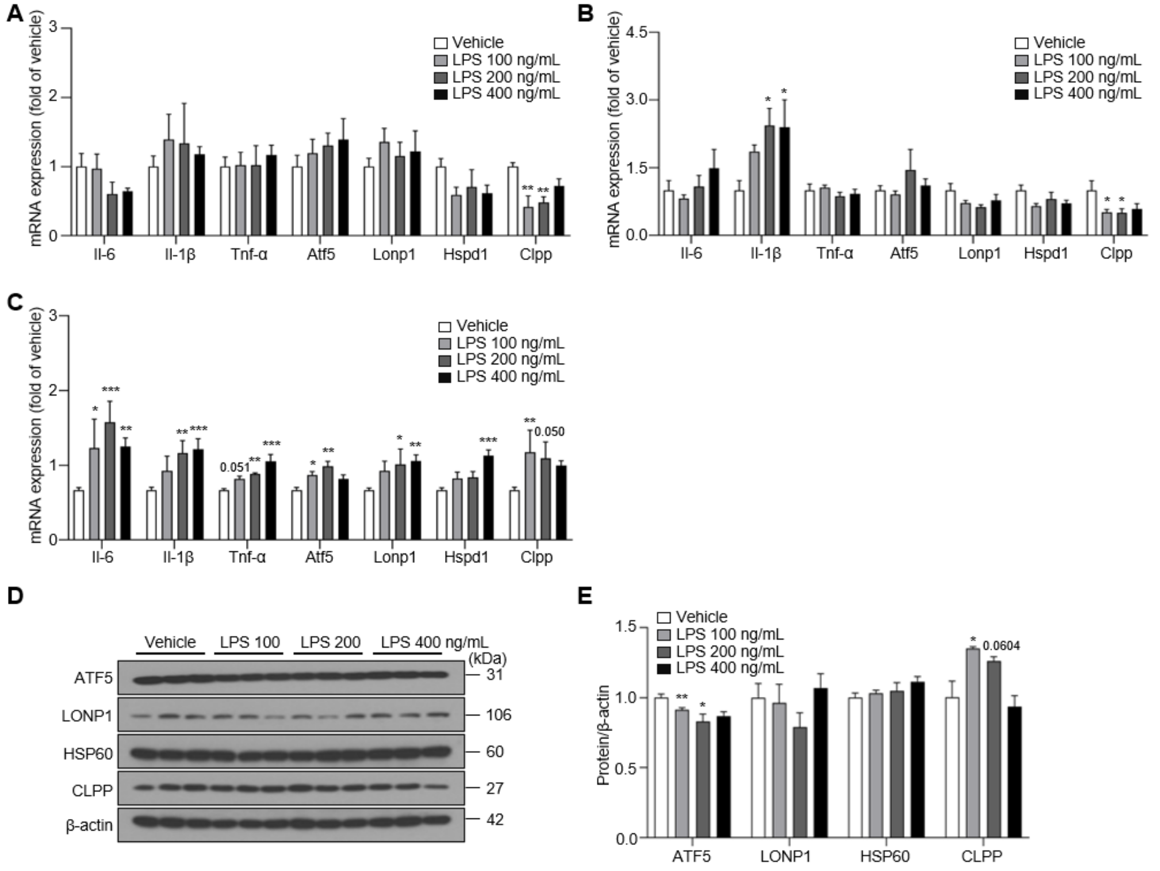

Supplementary Figure S1. The effect of LPS treatment on the production of *Il-6*, *Il-1β*, *Tnf-α*, *Atf5*, *Lonp1*, *Hspd1* and *Clpp* in BV-2 cells by time- and dose-dependent manner. (**A**) The expression of *Il-6*, *Il-1β*, *Tnf-α*, *Atf5*, *Lonp1*, *Hspd1* and *Clpp* in LPS-treated BV-2 cells at 6 h. (**B**) The expression of *Il-6*, *Il-1β*, *Tnf-α*, *Atf5*, *Lonp1*, *Hspd1* and *Clpp* in LPS-treated BV-2 cells at 12 h. (**C**) The expression of *Il-6*, *Il-1β*, *Tnf-α*, *Atf5*, *Lonp1*, *Hspd1* and *Clpp* in LPS-treated BV-2 cells for 24 h. (**D**) The expression of ATF5 and UPRmt molecules (LONP1, HSP60 and CLPP) in LPS-treated BV-2 cells for 24 h. (**E**) The intensity value of ATF5 and UPRmt molecules was shown. Data are presented as mean ± SEM of three independent experiments (* $p < 0.05$, ** $p < 0.01$, *** $p < 0.001$ compared to vehicle).

Supplementary Figure S2

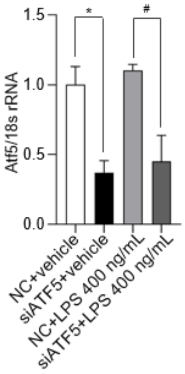

Supplementary Figure S2. The decrease of *Atf5* by siATF5 with or without LPS treatment. The mRNA expression of *Atf5* was measured in BV-2 cells treated with NC or siATF5 with vehicle or LPS. Data are presented as mean ± SEM of three independent experiments (* $p < 0.05$ compared to NC+vehicle, # $p < 0.05$ compared to NC+LPS 400 ng/mL).

Supplementary Figure S3

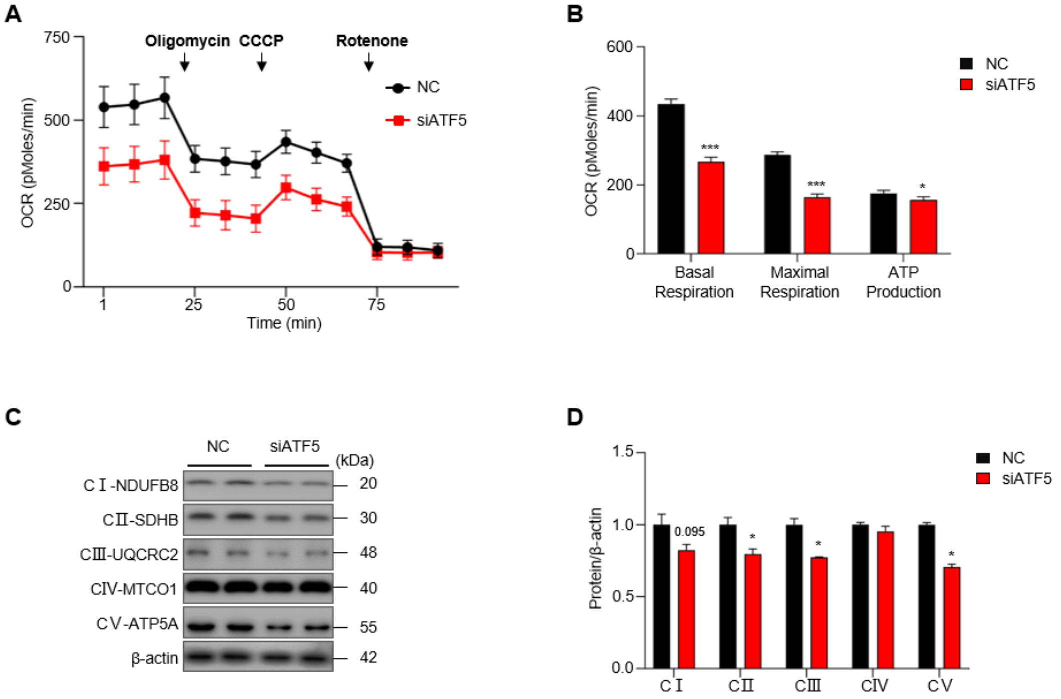

Figure S3. Treatment of siATF5 decreases the mitochondrial OXPHOS in BV-2 cells for 24 h. (A) Oxygen consumption rate (OCR) was measured in BV-2 cells treated with NC or siATF5 at 24 h. (B) The basal respiration, maximal respiration and ATP production were calculated by XF24 analyzer software. (C) The expression of mitochondrial complexes I, II, III, IV and V in NC- or siATF5-treated BV-2 cells. (D) The

intensity value of mitochondrial complexes was shown. Data are presented as mean ± SEM of three independent experiments (* $p < 0.05$, ** $p < 0.01$, *** $p < 0.001$ compared to NC).

Supplementary Table S1. Primers used for qPCR

Gene	Forward (5`→3`)	Reverse (5`→3`)
Atf5	ACCGCAAGCAAAAGAAGAGA	CAGCCTGGACCTGTACCCTA
Lonp1	GACAGAGAACCCGCTAGTGC	CTCAGTGGTTCTGGGATGGT
Hspd1	GAGCTGGGTCCCTCACTCG	AGTCGAAGCATTTCTGCGGG
Clpp	GCCATTCACTGCCCAATTCC	TGCTGACTCGATCACCTGTAG
Il-6	ACAACCACGGCCTTCCCTACTT	CACGATTTCCCAGAGAACATGTG
Il-1β	TGACGGACCCCAAAAGATGA	AAAGACACAGGTAGCTGCCA
Tnf-α	ACGGCATGGATCTCAAAGAC	AGATAGCAAATCGGCTGACG
18s rRNA	CGACCAAAGGAACCATAACT	CTGGTTGATCCTGCCAGTAG

Supplementary Table S2. Antibodies used for qPCR

Name	Source	Identifier
Total OXPHOS	Abcam	ab110413
ATF5	Abcam	ab184923
LONP1	Abcam	ab103809
HSP60	Abcam	ab46798
CLPP	Abcam	ab124822
β-actin	Santa Cruz Biotechnology	sc-47778
